# Supplementary material for: Histone Acetyltransferase GCN5 Affects Auxin Transport during Root Growth by Modulating Histone Acetylation and Gene Expression of PINs
Source: Plants (Basel). 2022 Dec 17;11(24):3572. doi: 10.3390/plants11243572 (PMC9781282; doi:10.3390/plants11243572)
Supplement: Supplementary file 1 [file plants-11-03572-s001.zip › plants-2055618-supplementary.pdf]

Supplemental Table 1. List of primers used in this study

Experiment	Target	Primer sequence (5' – 3')
Genotyping	GCN5	AGTGGGGGCACACTCGTTTCAAATTATTC
		TTGAGATTTAGCACCAGATTGGAGACCTG
	gcn5-1	CATTTTATAATAACGCTGCGGACATCTAC
		TTGAGATTTAGCACCAGATTGGAGACCTG
	ADA2b	ACTCCTCACAAATGTGATCACCCATACCG
		CTCCATCTCCGCCAAGAGTTGCTCAG
	ada2b-1	CATTTTATAATAACGCTGCGGACATCTAC
		CTCCATCTCCGCCAAGAGTTGCTCAG
	mGFP	TCAAGGAGGACGGAAACATC
		AAAGGGCAGATTGTGTGGAC
Gene Expression	*At4G26410*	GAGCTGAAGTGGCTTCCATGA
		GGTCCGACATACCCATGATCC
	PIN1	TAAGGTGATGCCACCAACAA
		AGCTGGCATTTCAATGTTCC
	PIN2	TGCCAACGATAATGAGTGGA
		ATTTTCCGCACGCAATAATC
	PIN3	GAGCACCTGACAACGATCAAG
		TCCACTTGCTGGATGAGCTAC
	PIN4	TGCTAAGGAGATTCGGATGG
		AAGACCGCCGATATCATCAC
	PIN7	TTCATCCCGCAATCTTGAGT
		ATCCTCTTCAGCCAAGCAGA
ChIP-qPCR	*PIN1* PR	GCACCCATCAACCACCATTTT
		AGTGTGTGTGATGTAATTTTGATTGA
	PIN1 CR	CTTTGGTCCTGGAGAAGCTG
		GGACCACCGTCTTCTTCGTA
	PIN3 PR	TTGCTCACTCCACACACACA
		AACCGTTAAGCAACGGTGTC
	PIN3 CR	CAAGTGGAGATTTCGGAGGA
		GGCGTCTTTTGGTCTCTCTG
	PIN4 PR	CAACAGTGGCTTGGACCTAA
		TGGTACAAGAAGACAGCCACA
	PIN4 CR	GTGATGATATCGGCGGTCTT
		TTCAGCCCTGCTGTAGCTTT
